# A potato STRUBBELIG-RECEPTOR FAMILY member, StLRPK1, associates with StSERK3A/BAK1 and activates immunity

**DOI:** 10.1093/jxb/ery310

**Published:** 2018-08-22

**Authors:** Haixia Wang, Yanlin Chen, Xingtong Wu, Zongshang Long, Chunlian Sun, Hairong Wang, Shumei Wang, Paul R J Birch, Zhendong Tian

**Affiliations:** 1 Key Laboratory of Potato Biology and Biotechnology, Ministry of Agriculture, Huazhong Agricultural University, Wuhan, China; 2 Key Laboratory of Horticultural Plant Biology, Ministry of Education, Huazhong Agricultural University, Wuhan, China; 3 Division of Plant Sciences, School of Life Science, University of Dundee, James Hutton Institute, Errol Road, Invergowrie, Dundee, UK

**Keywords:** Late blight, MAPK cascade, PAMP-triggered immunity, receptor-like kinases, StLRPK1, STRUBBELIG-RECEPTOR FAMILY

## Abstract

Plant *STRUBBELIG (SUB)-RECEPTOR FAMILY* (*SRF*) genes encode putative leucine-rich repeat transmembrane receptor-like kinases. SRFs have been reported to play essential roles in tissue morphogenesis in many plant organs. Here, we show that a potato *SRF* family gene, *StLRPK1*, is involved in plant immunity. StLRPK1 is located at the cell plasma membrane and is strongly induced by culture filtrate from *in vitro* growth of the late blight pathogen *Phytophthora infestans*. Overexpression of *StLRPK1* in stable transgenic potato or ectopic expression in *Nicotiana benthamiana* plants enhances *P. infestans* disease resistance, whereas RNA interference (RNAi) of *StLRPK1* in potato decreases disease resistance. We found that StLRPK1 constitutively interacts with a pivotal co-receptor, SERK3A/BAK1, which plays a central role in plant immunity. Virus-induced gene silencing of *SERK3A/BAK1* in *N. benthamiana* lines expressing *StLRPK1* attenuated *P. infestans* resistance, indicating that SERK3A/BAK1 is required for *StLRPK1*-mediated immunity. Finally, we show that StLRPK1-triggered late blight resistance depends on the mitogen-activated protein kinase kinase MEK2 and mitogen-activated protein kinase WIPK. We propose a model in which StLRPK1 associates with SERK3A/BAK1 to positively regulate plant immunity to *P. infestans* through a MAPK cascade. These data provide new insights into our understanding of SRF function in plant immunity.

## Teaser

The potato STRUBBELIG-RECEPTOR FAMILY protein StLRPK1 associates with StSERK3A/BAK1 to positively regulate potato and *Nicotiana benthamiana* immunity through a MAPK signaling pathway.

## Introduction

During their lifespan, plants must adapt to their environment, and so require mechanisms for sensing their surroundings and responding appropriately (Belkhadir *et al.*, 2014; Wolf, 2017). Living organisms sense and conduct signals through cell surface receptors. In plants, signal transduction is often initiated by receptor-like kinases (RLKs). The largest group of plant RLKs Ais the leucine-rich repeat RLK family (LRR-RLK) (Liu *et al.*, 2017). An expanded family of more than 200 LRR-RLKs has been reported in Arabidopsis (Shiu and Bleecker, 2001). Based on the phylogenetic relationships of kinase domains and the arrangements of LRR motifs, LRR-RLK proteins were classified into 15 groups in Arabidopsis (Shiu and Bleecker, 2001). Recently, plant LRR-RLKs have been expanded to 19 subfamilies (Liu *et al.*, 2017). LRR-RLKs are the largest group of RLKs in plants and play crucial roles in many processes during a plant’s life cycle, development, physiology, and immunity (Belkhadir *et al.*, 2014).

Plants have evolved a wide range of cell surface-resident RLKs and receptor-like proteins (RLPs) that detect conserved microbe-associated molecular patterns (MAMPs), leading to the activation of pattern-triggered immunity (PTI). Pattern recognition receptors (PRRs) FLAGELLIN-SENSING 2 (FLS2) and ELONGATION FACTOR-TU (EF-Tu) RECEPTOR (EFR) perceive bacterial flagellin and EF-Tu, respectively (Zipfel *et al.*, 2006; Chinchilla *et al.*, 2007). Pep is perceived by the Arabidopsis PEPR1/2 (Yamaguchi *et al.*, 2010), whereas chitin is perceived by Arabidopsis LysM-RK LYK5 and rice LysM-RP CEBiP (CHITIN OLIGOSACCHARIDE ELICITOR BINDING PROTEIN) (Kaku *et al.*, 2006; Shimizu *et al.*, 2010; Cao *et al.*, 2014). RECEPTOR-LIKE PROTEIN 23 (RLP23) perceives secreted NECROSIS-AND ETHYLENE-INDUCING PEPTIDE 1 (NEP1)-LIKE PROTEINS (NLPs) from various plant-associated microorganisms (Albert *et al.*, 2015). The csp22 peptide derived from bacterial cold shock protein is perceived by the tomato LRR-RK CORE and tobacco LRR-RP NbCSPR (Saur *et al.*, 2016; Wang *et al.*, 2016). The oomycete *Phytophthora* elicitin INF1 is recognized by potato ELICITIN RESPONSE (ELR) (Du *et al.*, 2015). After ligands have been recognized by bona fide LRR-RLK receptors, the signaling pathways activated by RLKs and RLPs often require a small set of co-receptors, called somatic embryogenesis receptor kinases (SERKs), often complexed via ligand-induced heterodimerization and transphosphorylation (Chinchilla *et al.*, 2009; Ma *et al.*, 2016; Tang *et al.*, 2017). The plant LRR-RLK BRI1-ASSOCIATED RECEPTOR KINASE 1 (BAK1), also known as SERK3A/BAK1, has been identified as a co-receptor in diverse signaling receptorsomes, such as the brassinosteroid receptor BRASSINOSTEROID INSENSITIVE 1 (BRI1) and the immune PRRs FLS2, EFR, and ELR (Nam and Li, 2002; Zipfel *et al.*, 2006; Chinchilla *et al.*, 2007; Du *et al.*, 2015).

In Arabidopsis, the monophyletic LRR-V family of RLKs consists of nine different genes, one of which is *STRUBBELIG* (*SUB*). The LRR-V family has been called the STRUBBELIG-RECEPTOR FAMILY (SRF); it includes SUB (SRF9) and the family members SRF1 to SRF8 (Eyüboglu *et al.*, 2007; http://www.arabidopsis.org/browse/genefamily/lrrv.jsp). SRFs contain a signal peptide, a SUB domain (an amino-terminal region of about 59 residues that is conserved in SRF members), six LRRs, a proline-rich region, a transmembrane domain, and a putative C-terminal cytoplasmic kinase domain (Chevalier *et al.*, 2005; Vaddepalli *et al.*, 2011). Biochemical and genetic data indicate that, although the kinase domain is essential for SUB function, enzymatic phosphotransferase activity is not (Vaddepalli *et al.*, 2011). Thus, SUB is likely a so-called enzymatically inactive kinase. Global gene expression analysis has shown that several Arabidopsis *SRF* transcripts are present in a broad pattern, including cauline leaves, flowers, siliques, stems, roots, and seedlings (Eyüboglu *et al.*, 2007). *SRF4* and *SRF5* show highly pronounced expression in mature pollen. *SUB* (*SFR9*) displays increased expression levels in shoot apices at the bolting stage. *SRF1* and *SRF3* expression profiles show inductions or repressions in experiments investigating programmed cell death, tumor development, control of lignification, and pectin biosynthesis. *SRF6* was strongly induced in plants exposed for a prolonged time (3 h) to heat stress and infections, with fungi inducing arbuscular mycorrhizal symbiosis. *SRF7* and *SRF8* showed prominent expression in experiments analyzing the effects of sulfate limitation on transcription. Both *SRF6* and *SRF7* showed elevated transcript levels in experiments involving brassinosteroid treatments (Eyüboglu *et al.*, 2007). This indicates that an increase in *SRF* transcripts occurs in response to many environmental stimuli. In Arabidopsis, SUB is essential for several developmental processes, including the formation of carpels, petals, ovules, and root hair patterning. The mutant *sub* phenotype suggests that SUB affects the formation and shape of several organs by influencing cell morphogenesis, the orientation of the division plane, and cell proliferation. Ovules of *sub* mutants show frequent defects in the initiation and outgrowth of the outer integument. *sub* mutants exhibit twisted stems, petals, and carpels/siliques. In addition, *sub* mutations lead to a randomization of root hair patterning (Chevalier *et al.*, 2005; Kwak *et al.*, 2005; Yadav *et al.*, 2008; Fulton *et al.*, 2009). SUB also controls stem and floral organ shape (Chevalier *et al.*, 2005; Fulton *et al.*, 2009; Vaddepalli *et al.*, 2011). In addition, SRF4 plays a role in the regulation of leaf size; SRF4 or SRF7 were proposed to be involved in male sterility; SRF7 may be involved in primary cell wall biosynthesis; and SRF8 may contribute to sterol biosynthesis (Eyüboglu *et al.*, 2007). SRF3 has been reported to play a role in genetic incompatibility in Arabidopsis, a phenotype linked closely to an RPP1-mediated plant pathogen immune response (Alcázar *et al.*, 2010).

Although there are *SRF* ortholog genes in the genomes of other plant species, little is known about the functions of such receptors. Previously, we isolated a potato receptor-like kinases gene, *StLRPK1*, which was induced during infection by the oomycete *Phytophthora infestans*, the causal agent of potato late blight (Wu *et al.*, 2009). StLRPK1 shares conserved domains with Arabidopsis SRFs. Here, we provide further insight into the role of *StLRPK1* in plant immunity. StLRPK1 was located at the cell plasma membrane and it was strongly induced by *P. infestans* culture filtrate (CF), which could be regarded as a cocktail of *Phytophthora* MAMPs that induce PTI in Solanaceae (McLellan *et al.*, 2013). The overexpression of *StLRPK1* in transgenic potato or ectopic expression in *Nicotiana benthamiana* strongly enhanced resistance to *P. infestans*, indicating that StLRPK1 is a positive regulator of immunity to *P. infestans* in potato and *N. benthamiana*. Moreover, we confirmed that StLRPK1 interacts with a pivotal co-receptor, SERK3A/BAK1, during immunity and that this is essential for *StLRPK1*-mediated resistance in *N. benthamiana*. Finally, we provide evidence that StLRPK1-triggered resistance to *Phytophthora* is dependent on the mitogen-activated protein kinase kinase (MAP2K) MEK2 and the mitogen-activated protein kinase (MAPK) WIPK. We propose a model in which StLRPK1 associates with SERK3A/BAK1 to positively regulate plant innate immunity to *P. infestans* through a MAPK cascade. To our knowledge, this is the first report of an SRF associating with co-receptor SERK3A/BAK1 to activate plant immunity.

## Materials and methods

### Constructs

The coding region of *StLRPK1* was digested from the pMD18-T-StLRPK1 construct by using *Hin*dIII/*Bam*HI enzymes and inserted into pBI121. For the RNAi vector, a non-conserved fragment of *StLRPK1* was amplified by the attB1-StLRPK1-RNAi-F and attB2-StLRPK1-RNAi-R primers (see Supplementary Table S1 at *JXB* online) and recombined into the entry vector pDONR201 using BP clonase (Invitrogen), followed by recombination into pHellsgate8 using LR clonase (Invitrogen). pBI121-*StLRPK1* and pHellsgate8-*StLRPK1* vectors were transformed into *Agrobacterium tumefaciens* strain LBA4404 by electroporation and cultured on YEB medium containing appropriate antibiotics. StLRPK1-GFP, StSERK3A-cMyc, and NbSERK3A-cMyc were cloned from potato and *N. benthamiana* by PCR with gene specific primers (Supplementary Table S1) modified to contain the Gateway (Invitrogen) attB recombination sites. The PCR products were recombined into pDONR201 (Invitrogen) to generate entry clones, followed by recombination into pK7FGWT7, PGWB17, and PGWB20 (Nakagawa *et al.*, 2007), respectively, by using LR clonase (Invitrogen), Vectors were then transformed into *A. tumefaciens* strain GV3101. cMyc-StBSL1-PGWB18 was described by Saunders *et al* (2012).

### Confocal microscopy

*Agrobacterium tumefaciens* strain GV3101 containing the fusion protein constructs was grown overnight in YEB medium containing selective antibiotics at 28 °C, pelleted, resuspended in inﬁltration buffer [10 mM 2-(*N*-morpholino)ethanesulfonic acid, 10 mM MgCl_2_, and 200 mM acetosyringone], and pressure infiltrated into leaves of 4-week-old *N. benthamiana* or transgenic *N. benthamiana* line CB173 (expressing a plasma membrane marker gene mOrang-Lti6; Wang *et al.*, 2017). StLRPK1-GFP and mRFP (cytoplasmic marker) were also co-expressed in *N. benthamiana* leaves. Fluorescence was observed at 2 days post-infiltration (dpi) using a Nikon A1R confocal microscope and water dipping lenses. GFP fluorescence was observed with excitation at 488 nm and emissions were collected between 500 and 530 nm. Imaging of mOrange and mRFP fluorescent proteins was performed using excitation at 561 nm and emissions were collected between 600 and 630 nm. Image processing for the figures was conducted with Adobe Photoshop CS5 and Adobe Illustrator CS6.

### Plant transformation and growth conditions

*Agrobacterium tumefaciens* containing the overexpression vector pBI-35S-*StLRPK1* and pHellsgate8-*StLRPK1* was transformed into a Chinese potato cultivar, ‘E-potato-3’ (E3), by microtuber disc transformation, as described by Tian *et al.* (2015). Transgenic plants were selected on Murashige and Skoog (MS) medium containing kanamycin and confirmed by PCR with the gene-specific primers of *NPTII*. The expression level of the transgene was analyzed by quantitative real-time reverse transcription PCR (qRT-PCR) (primers are shown in Supplementary Table S1). The plantlets were maintained and propagated by growing single nodes on MS medium in growth chambers at 22 °C with a 16 h photoperiod. For *N. benthamiana,* leaf discs were used as explants for transformation using *A. tumefaciens* containing pBI121-35S: *StLRPK1*. All other conditions were the same as for potato transformation. Self-pollinated seeds from transgenic plants were collected for further use.

Transgenic and E3 potato plantlets were propagated on MS medium supplemented with 4% sucrose and 0.7% agar, and raised in a climate room under controlled conditions (16/8 h light/dark cycle at 22 °C). Four-week-old transgenic potato lines were transferred and grown in individual pots with general-purpose compost in the greenhouse under normal conditions. Seven-week-old potato plants were used for *P. infestans* inoculation. Homozygous transgenic *N. benthamiana* seeds were collected and sown in general-purpose compost; 2-week-old seedlings were transplanted into individual pots and were grown in a growth chamber with a 16/8 h light/dark cycle at 22–24 °C and 70% humidity. *N. benthamiana* plants at 5–6 weeks old were used for agroinfiltration and *P. infestans* inoculation.

### Culture filtrate and flg22 treatment

*Phytophthora infestans* CF was prepared by the inoculation of sterile Plich media (0.5 g KH_2_PO_4_, 0.25 g MgSO_4_.7H_2_O, 1 g asparagine, 1 mg thiamine, 0.5 g yeast extract, 10 mg β-sitosterol, 25 g glucose) with *P. infestans* strain 88069 for *N. benthamiana* treatment, or with mixture of the isolates HB09-14-2 and HB09-21 for potato treatment. Inoculated media were incubated in darkness at room temperature for 2–3 weeks before being centrifuged to remove mycelium. The synthetic 22-amino-acid flg22 peptide (QRLSSGLRINSAKDDAAGLAIS) was dissolved at a concentration of 40 µM in sterile distilled water before infiltration of leaves in the same manner. CF or flg22 solution was pressure infiltrated into leaves using a 1 ml plastic syringe (without a needle) until the liquid spread dimeter reached ~1.5 cm. The treated leaves were collected at 0, 0.5, 1, 3, and 6 h for RNA extraction.

### *P. infestans* inoculation and determination of biomass

*Phytophthora infestans* isolates HB09-14-2 and HB09-21 (Tian *et al.*, 2015), with different pathogenicity, were used for inoculating potato leaves. *P. infestans* strain 88069 was used for inoculation of *N. benthamiana* plants. *P. infestans* isolates were routinely grown on rye agar medium supplemented with 2% sucrose at 18 °C in the dark. *P. infestans* sporangia were collected as described by Champouret *et al.* (2009). Sporangia were quantified using a hemocytometer, and the inoculation concentration was adjusted to 1 × 10^5^ sporangia ml^−1^ for potato plants and 8 × 10^4^ sporangia ml^–1^ for *N. benthamiana* plants. Droplets (10 µl) were inoculated on to the abaxial side of detached leaves, which were stored on moist tissue in sealed boxes. Lesions were measured at 5 dpi for transgenic potato lines and 6 dpi for transgenic *N. benthamiana* plants. Biomass assays were performed as described previously (Tian *et al.*, 2015). qRT-PCR was applied to determine the growth of *P. infestans* on potato leaves; *P. infestans*-specific primers used for amplification and detection are shown in Supplementary Table S1. *P. infestans* hyphae on infected potato leaves were stained with trypan blue as described by He *et al.* (2015).

### Co-immunoprecipitation and western blot

*Agrobacterium tumefaciens* strain GV3101 containing the fusion protein constructs was inﬁltrated into *N. benthamiana* leaves. Four leaf discs for each sample were collected at 48 h post-infiltration and immediately frozen in liquid nitrogen. Protein extraction was carried out by incubating ground leaf tissue samples in 400 µl extraction buffer (10% glycerol, 25 mM Tris, pH 7.5, 150 mM NaCl, 10 mM dithiothreitol, 0.15% Nonidet P40, 1 mM phenylmethylsulfonyl fluoride, protease inhibitors with 1 mM EDTA) on ice for 0.5 h, followed by centrifugation at 12470 *g* at 4 °C for 10 min. A 40 µl aliquot of supernatant was removed and 40 µl 2×SDS sample loading buffer was added. The protein samples were boiled at 95 °C for 10 min for Western blots. Then, 20 µl of equilibrated GFP-Trap beads was incubated in the remaining supernatant at 4 °C for 2 h with constant mixing in a tube rotator. The GFP beads were magnetically separated and then washed with 500 µl of washing buffer (10% glycerol, 25 mM Tris, pH7.5, 150 mM NaCl, 1 mM phenylmethylsulfonyl fluoride, protease inhibitors with 1 mM EDTA) three times. GFP beads were eluted with 50 µl 2×SDS sample loading buffer and the eluate was incubated at 95 °C for 10 min. Protein samples were separated on 12% polyacrylamide gels and transferred to polyvinylidene fluoride membranes (Bio-Rad, Hercules, CA, USA) according to the manufacturer’s instructions. The membranes were blocked in 4% milk in 1×PBS with 0.1% Tween (1×PBST) by shaking for 1 h at room temperature and were then incubated overnight with a polyclonal GFP or cMyc antibody (MBL, Nagoya, Japan) at 1:2000 and in 4% milk 1×PBST. A secondary incubation with anti-mouse IgGHRP (MBL, Nagoya, Japan) at 1:5 000 was carried out for 1 h.

### Virus-induced gene silencing in *N. benthamiana*

Virus-induced gene silencing (VIGS) was performed by using a tobacco rattle virus (TRV) vector (Liu *et al.*, 2002). A TRV construct expressing GFP was used as a control TRV-SERK3A/BAK1 construct, as described previously (Heese *et al.*, 2007). Primers for TRV2-*NbSERK3A/BAK1*, TRV2-*NbMEK1*, TRV2-*NbMEK2*, and TRV2-*NbWIPK* are shown in Supplementary Table S1. *Agrobacterium tumefaciens* GV3101 containing a mixture of TRV1 and each TRV2-gene construct at OD_600_=0.5 were infiltrated into the upper leaves of four-leaf-stage *N. benthamiana* plants. Systemic leaves were detached and analyzed by qRT-PCR for silencing efficiency, and used for *P. infestans* colonization assays 2–3 weeks later as described previously by McLellan *et al.* (2013).

### Gene expression analysis

Total RNA was extracted from plant tissue by using the EASY spin plant RNA extraction kit (Aidlab, Beijing, China) according to the manufacturer’s instructions, including the on-column DNase treatment. RNA was quantified using a Nanodrop 1000 (Thermo Fisher Scientific) and cDNA synthesized using Hiscript Reverse Transcriptase (Vazyme, Nanjin, China) and oligo(dT) primers (Vazyme). qRT-PCR was performed using Bio-Rad SYBR Green Supermix, and PCR parameters were as follows: 95 °C for 5 min (first cycle); 40 cycles of 95 °C for 10 s, 58 °C for 10 s, and 72 °C for 30 s; and a final cycle of 72 °C for 5 min. PCRs were performed in triplicate with a Bio-Rad CFX Connect^TM^ Real-Time Detection System (Bio-Rad, Hercules, CA, USA). Gene expression levels were calculated by a comparative ΔΔCt method as described in the manufacturer’s instructions for the CFX Connect^TM^ Real-Time Detection System. All primers, including internal controls used for measurement of transcript abundance, are shown in Supplementary Table S1.

## Results and discussion

### StLRPK1 belongs to the STRUBBELIG-RECEPTOR FAMILY (SRF) subfamily

**Fig. 1.**
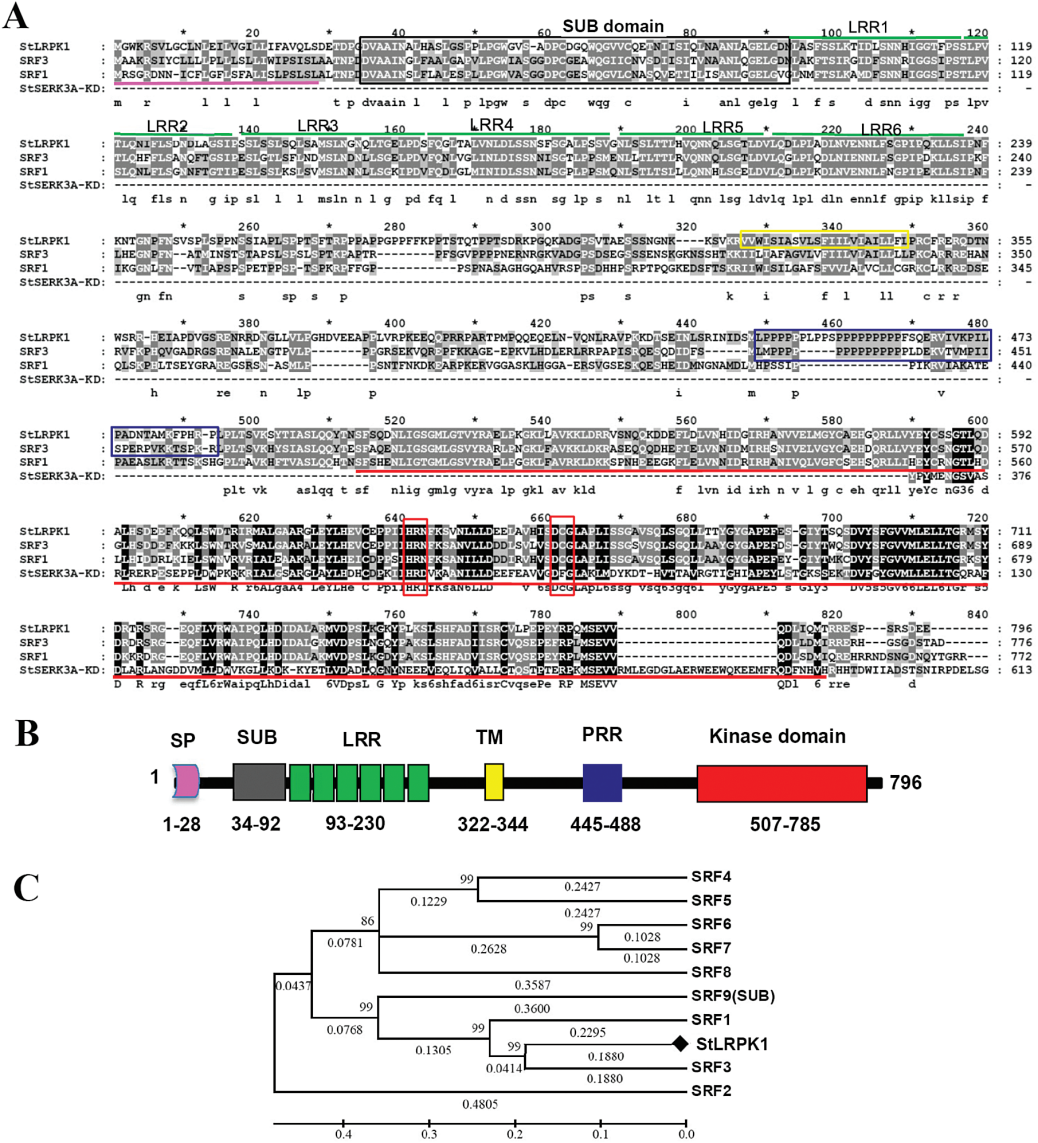
Protein alignment of StLRPK1 with Arabidopsis SRF family protein sequences. (A) Alignment of the amino acid sequences of SRF full-length proteins and the StSERK3A kinase domain. Full conservation across the kinase domain alignment is marked by black columns and partial conservation by gray columns. The predicted signal peptide sequences and the kinase subdomain are underlined with thick pink and red lines, respectively. The SUB domains, the transmembrane domains, and the proline-rich regions are marked with black, yellow and blue rectangles, respectively. The six LRRs are marked with green lines above each LRR region. Two red rectangles indicate the regions containing two typical HxD and DFG motifs in active kinases, corresponding to the VI and VIIa subdomains of the StSERK3A/BAK1 kinase domain. (B) Schematic representation of the StLRPK1 structure. LRR, leucine-rich repeat; PRR, proline-rich region; SP, signal peptide; SUB, SUB domain; TM, transmembrane domain. (C) Phylogenetic tree of the SRF family. A maximum likelihood tree obtained using the amino acid sequences of the potato StLRPK1 and Arabidopsis SRF members as input. The branch support values are indicated. The protein GenBank accession numbers for alignments are as follows: StLRPK1 (EU049848), Arabidopsis SRF1 (AY518286), SRF2 (AY518287), SRF3 (AY518288), SRF4 (AY518289), SRF5 (AY518290), SRF6 (AY518291), SRF7 (AY518292), SRF8 (AY518293), SRF9 (AF399923, SUB), and StSERK3A/BAK1 (AGT21432.1).

RLKs are a prominent class of cell surface receptors that regulate many aspects of plant development, hormone signaling, and immunity (Antolín-Llovera *et al.*, 2012). We previously isolated a receptor-like kinase gene, *StLRPK1*. StLRPK1 protein shares conserved domains with SRF members in *Arabidopsis thaliana* (Wu *et al.*, 2009). Here, we compared StLRPK1 with the Arabidopsis SRF1 and SRF3 in detail. StLRPK1 contains all the typical conserved domains of SRFs (Fig. 1A, B). We compared putative SRF kinase domains with the potato StSERK3A/BAK1 kinase domain and found that they share low similarity. Interestingly, two typical His-x-Asp (HxD) and Asp-Phe-Gly (DFG) motifs found in active kinases (Zhang *et al.*, 2015) were replaced by HRN and DCG in the three SRF kinase domains (Fig. 1A). This indicates that StLRPK1 is likely an enzymatically inactive kinase. This is something that will be tested in the future.

Phylogenetic analysis was performed using the full-length amino acid sequence of the nine SRF members from Arabidopsis (SRF1 to SRF9) and StLRPK1. This showed that StLRPK1 is more closely related to SRF3 (AY518288) of the Arabidopsis SRF family (Fig. 1C). StLRPK1 shares high identity with predicted solanaceous SRF3-like proteins from tomato [XP_010323714.1, *Solanum lycopersicum* 758/796 (95%)], *Capsicum annuum* [XP_016580745.1, 716/796 (90%)], and *Nicotiana tomentosiformis* [XP_009625937.1, 680/797 (85%)]. In contrast, StLRPK1 shares only 76% (605/800) identity with a potato predicted SRF3 isoform X1 (XP_006350044.1) and 74% (595/800) identity with a tomato predicted SRF3 isoform X1 (XP_004251794.1), reflecting the possibility that SRF3 and SRF3-like proteins perform different functions in Solanaceae species. The alignment and conservation of seven SRF3-like protein sequences along with a predicted potato SRF3 isoform X1 protein from Solanaceae species are highlighted in Supplementary Fig. S1.

### *StLRPK1* responds to oomycete MAMP treatment and the protein localizes to the plasma membrane

**Fig. 2.**
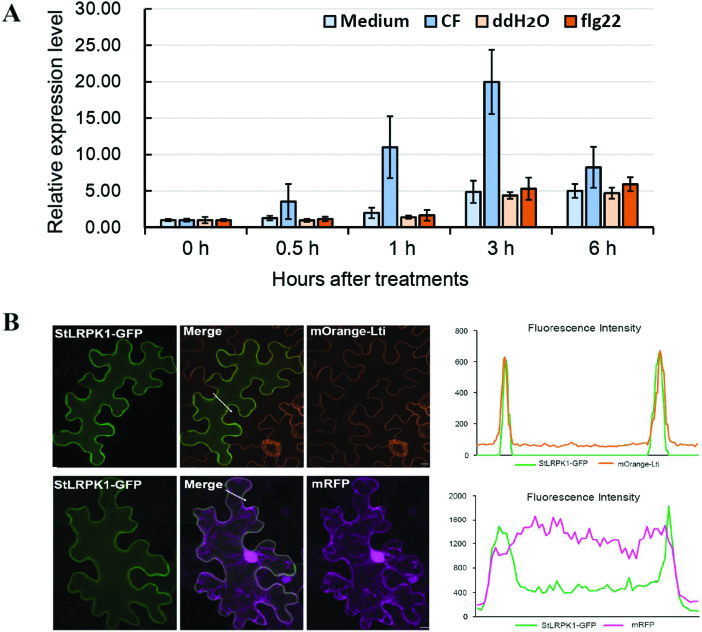
*StLRPK1* transcript abundance in response to flg22 and CF, and the localization of StLRPK1-GFP. (A) Expression of *StLRPK1* in response to flg22 and *P. infestans* culture filtrate (CF) treatment. Leaves on intact potato plants were pressure infiltrated with *P. infestans* CF or medium as a control, or flg22 or double-distilled H_2_O (ddH_2_O) as a control. Treated leaves were collected at 0, 0.5, 1, 3, and 6 h. qRT-PCR was performed to test the gene expression level. Data represent three biological replicates. (B) Representative confocal images of StLRPK1-GFP localization. Transient expression of StLRPK1-GFP in transgenic *N. benthamiana* expressing the plasma membrane (PM) marker mOrange-LTi6 or co-expression of StLRPK1-GFP with the cytoplasmic marker mRFP. From left to right: the green channel (StLRPK1-GFP), the merged channel, and the orange channel (mOrange-Lti6) or red channel (mRFP). The plots of fluorescence profiles (to the right of the confocal images), represented by the white arrows in the two merged images, indicate clear co-localization of StLRPK1-GFP with the PM marker but no co-localization with free mRFP. Scale bars=10 μm.

Wu *et al.* (2009) showed that *StLRPK1* was up-regulated in response to *P. infestans*. In this study, we tested whether *StLRPK1* was induced by either the bacterial MAMP flg22 or by *P. infestans* CF, which likely contains several *Phytophthora* MAMPs that induce PTI marker genes in potato (McLellan *et al.*, 2013). As shown in Fig. 2A, *StLRPK1* transcript abundance increased strongly in response to CF, but did not change with flg22 treatment, as compared with their corresponding controls (*P. infestans* liquid medium and H_2_O treatment, respectively) in the time-course of the experiment; this indicates that *StLRPK1* is involved in specific immune responses. To examine whether the predicted potato SRF3 isoform X1 (XP_006350044.1), which shares only 76% identity with StLRPK1, could be induced by CF, the expression levels were tested 1, 3, and 6 h after CF treatment. It was found that, unlike StLRPK1, the potato SRF3 isoform does not respond to CF, indicating that it is differentially expressed and therefore potentially has a different function (Supplementary Fig. S2). Alcázar *et al.* (2010) showed that SRF3 allelic variants were localized to the plasma membrane when transiently expressed in *N. benthamiana*. As a candidate SRF3-like homolog, StLRPK1 contains a transmembrane domain. To test whether StLRPK1 localizes to the plasma membrane, GFP was fused to the C-terminus of StLRPK1 to form StLRPK1-GFP. Transient expression of StLRPK1-GFP in transgenic *N. benthamiana* (carrying the plasma membrane marker mOrange-LTi6) showed clear co-localization with mOrange-LTi6 when examined by using confocal microscopy. In contrast, StLRPK1-GFP did not co-localize with free mRFP (cytoplasmic marker) in *N. benthamiana* (Fig. 2B). This supports the hypothesis that StLRPK1 may act at the level of MAMP recognition or signaling.

### Phenotypes of transgenic potato plants with altered *StLRPK1* expression

**Fig. 3.**
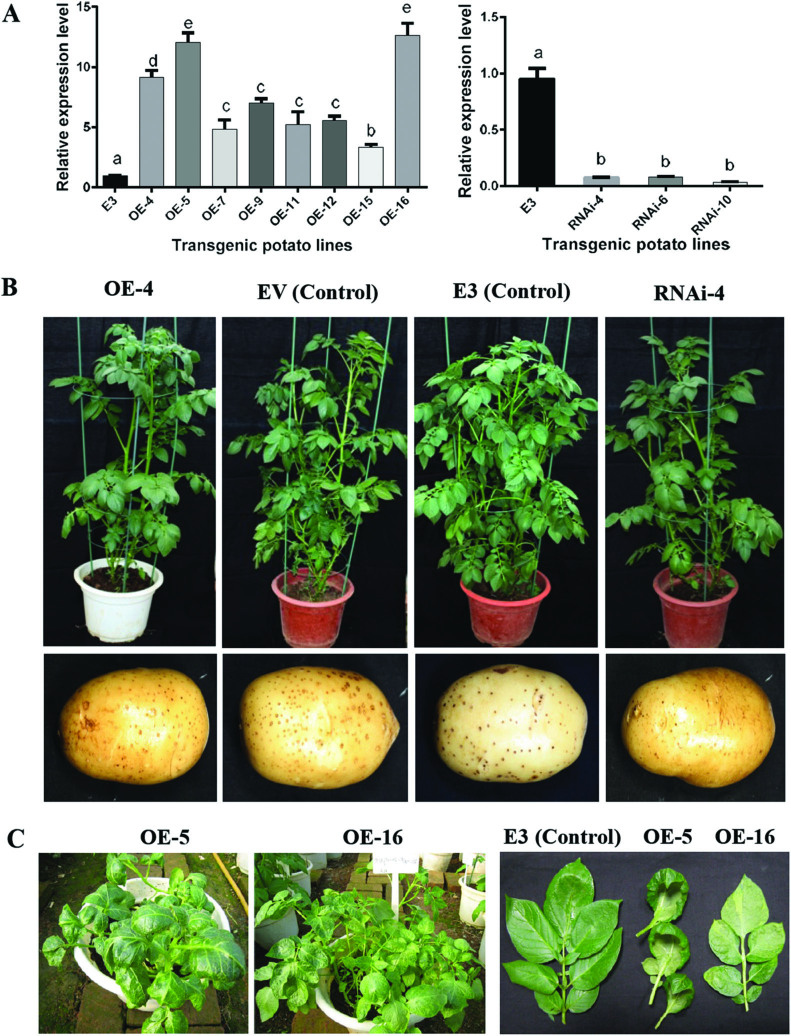
Representative phenotypes of *StLRPK1* overexpression (OE) and RNAi potato lines. (A) Target gene expression level in transgenic potato OE and RNAi lines. qRT-PCR was performed in three biological replicates. Statistical analysis was carried out using ANOVA with Tukey’s multiple comparisons test. Lower case letters denote statistically significant differences relative to the non-transgenic control line ‘E-potato-3’ (E3) (*P*<0.05). (B) Phenotype of 6-week-old *StLRPK1* OE and RNAi transgenic potato lines, E3 non-transgenic control plants, and EV (E3 35S: GUS-transgenic) control plants. (C) Abnormal leaf shape of two OE lines.

To investigate the function of StLRPK1, we made two constructs, pBI121-*StLRPK1* and pHellsgate8-*StLRPK1*. The pBI121-*StLRPK1* construct harbored the full-length gene of *StLRPK1* under the control of the CaMV 35S promoter. The pHellsgate8-*StLRPK1* construct contained two inverted repeats of the partial *StLRPK1* gene and was used for downregulating *StLRPK1* expression by RNAi. Overexpression and RNAi constructs were transferred into the susceptible Chinese potato variety E3 via *A. tumefaciens*–mediated stable transformation. In total, eight transgenic overexpression lines and three RNAi lines were produced. The transcript level in overexpression lines ranged from 4- to 15-fold higher than the level in control plants, while only ~10% of the wild-type transcript level was detected in the RNAi plants (Fig. 3A).

The *in vitro* plantlets of overexpression lines and RNAi lines showed no obvious phenotypic differences. The phenotypes of the field-grown plants were further observed; again, most overexpression lines and the three RNAi lines showed no obvious phenotypic changes compared with wild-type E3 during the growing season. The height, leaves, and tubers of transgenic plants of most lines did not differ from the wild type (Fig. 3B), indicating that, unlike many characterized SRF family members, StLRPK1 may not be involved in development. However, the two highest-level overexpression lines, OE-5 and OE-16, showed retarded growth and a dwarf phenotype. In addition, a cup-shaped single leaf grew on the stem of line OE-5, rather than a compound leaf (Fig. 3C). In contrast, a compound leaf was formed on the OE-16 line, although the size of the leaflets was smaller than that on the wild-type control. It is likely that high overexpression of StLRPK1, as observed in the OE-5 and OE16 lines, led to ‘off-target’ regulatory effects from the excessive protein levels.

### StLRPK1 positively regulates *P. infestans* resistance in potato and *N. benthamiana*

As reported by Wu *et al.* (2009), *StLRPK1* is induced in potato leaves by *P. infestans*. In this study, we found that *StLRPK1* was induced by *P. infestans* CF, which likely contains several oomycete MAMPs. This prompted us to investigate whether *StLRPK1* contributes to late blight resistance in potato.

**Fig. 4.**
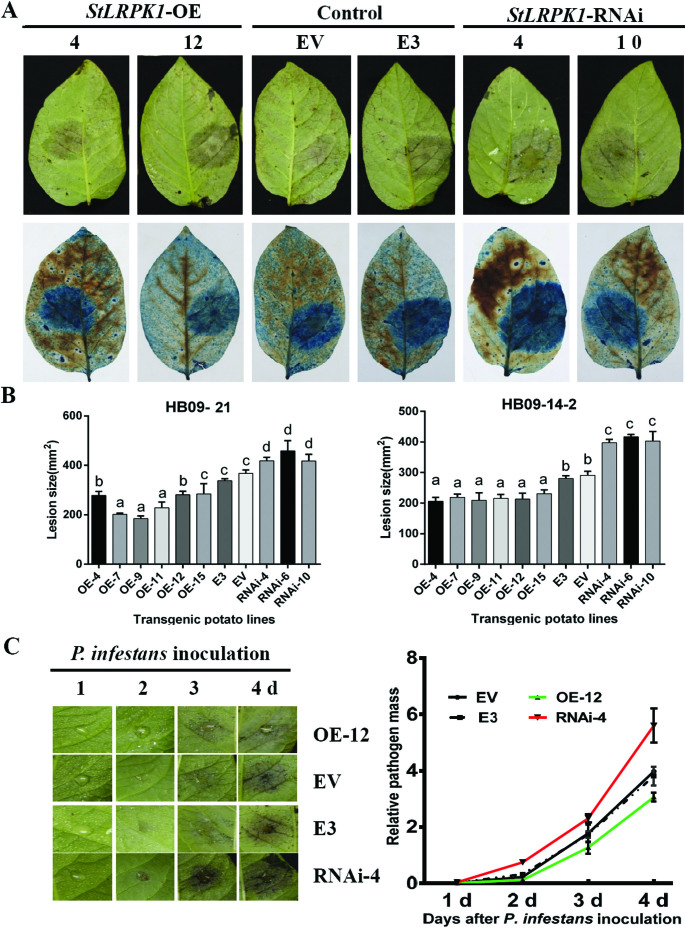
StLRPK1 positively regulates late blight resistance in potato. (A) Representative images of detached leaves of transgenic potato cv. ‘E-potato-3’ (E3) overexpressing (OE) or RNAi *StLRPK1*, and E3 and EV (35S:GUS transformant) controls infected with *P. infestans* and photographed at 5 dpi. The lower images are of leaves stained with trypan blue. (B) Average *P. infestans* lesion area (mm^2^) at inoculation sites on OE lines, RNAi lines, and E3 and EV controls measured at 5 dpi. Leaves were detached from 8-week-old potato plants and inoculated with suspensions of sporangia of *P. infestans* isolates HB09-21 and HB09-14-2 (10^5^ sporangia ml^−1^) separately. The total number of samples per line involved more than 60 leaves combined with three or four replicates from four plants of each line. Values labeled with different letters are statistically different by one-way ANOVA using pairwise multiple comparison procedures with the Holm–Sidak method (*P*<0.05); error bars show ±SD. (C) Representative image of *P. infestans* lesions development (left) and dynamic growth of the pathogen biomass (right) at infected sites on leaves of OE and RNAi lines, and E3 and EV controls, from 1 to 4 dpi. Biomass was calculated at infected sites using qRT-PCR. Error bars represent ±SE. Three biological replicates were performed, each combining five inoculation sites.

Two different *P. infestans* isolates were used to evaluate the resistance of transgenic lines. In each line, *P. infestans* colonization was measured as lesion size; disease lesions are strikingly apparent with trypan blue staining of the (Fig. 4A). Compared with the untransformed cultivar E3 and another transgenic line transformed with a 35S:GUS construct (EV), the lesion areas of three RNAi lines (4, 6 and 10) were significantly (*P*<0.01, one way ANOVA) larger than those in the controls. In contrast, the disease lesion areas in six overexpression lines were significantly smaller than those of the controls, apart from OE-15, where the lesion area caused by one of the *P. infestans* isolates was not significantly different from that in the two controls (Fig. 4B). qRT-PCR was performed to evaluate the biomass of *P. infestans* during pathogen colonization. The results showed that *P. infestans* mycelium biomass at inoculated sites was lower in a selected overexpression line and higher in a selected RNAi line compared with the E3 and EV controls after 3 dpi (Fig. 4C). In conclusion, overexpression lines showed smaller disease lesions and lower pathogen biomass, suggesting that StLRPK1 may positively regulate late blight resistance in potato.

**Fig. 5.**
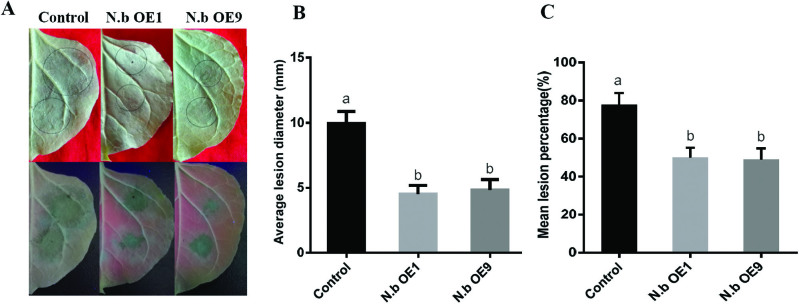
Ectopic overexpression of *StLRPK1* in *N. benthamiana* enhances late blight resistance. (A) Representative image of *P. infestans* lesions at 6 dpi on leaves of wild-type control *N. benthamiana* and two transgenic homozygous lines ectopically overexpressing *StLRPK1*. The lower photographs were taken under UV light. *P. infestans* isolate 88069 sporangia were used to inoculate leaves. (B, C) Mean *P. infestans* lesion diameter (B) and mean lesion percentage (C) on control and transgenic *N. benthamiana* leaves, measured at 6 dpi (three replicates, 30 leaves per replicate). Values labeled with different letters are statistically different by one-way ANOVA using pairwise multiple comparison procedures with the Holm–Sidak method (*P*<0.01). Error bars represent ±SD.

*Nicotiana benthamiana*, as a model host plant for *P. infestans*, has been extensively used to investigate pathogen and host gene functions in *P. infestans*–plant interactions (McLellan *et al.*, 2013; Whisson *et al.*, 2016; Wang *et al.*, 2017). Transgenic *N. benthamiana* plants ectopically overexpressing *StLRPK1* were obtained. Two independent homozygous *35S: StLRPK1* lines were used further to test late blight resistance. Purified lines came from self-pollination and were confirmed by kanamycin resistance selection and PCR tests (Supplementary Fig. 3). We found that ectopically overexpressing *StLRPK1* in *N. benthamiana* enhanced late blight resistance, as indicated by significantly (*P*<0.001, based on ANOVA) smaller disease lesion diameters and lower infection percentage (i.e. the percentage of inoculated leaves forming an infection lesion) compared with that of untransformed *N. benthamiana* plants (Fig. 5). Taken together, overexpressing *StLRPK1* in potato and in *N. benthamiana* reduced *P. infestans* colonization, demonstrating that StLRPK1 positively regulates immunity in these solanaceous plants.

### StLRPK1 interacts with StSERK3A/BAK1 and NbSERK3A/BAK1 *in planta*

**Fig. 6.**
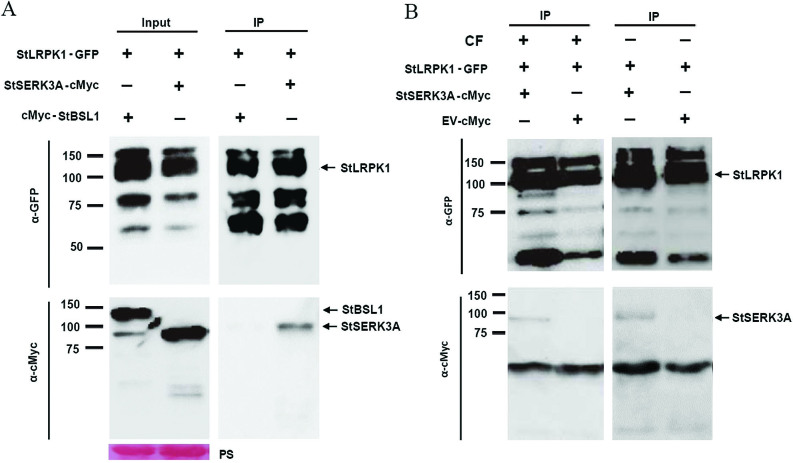
StLRPK1 interacts with StSERK3A *in planta*. (A) Immunoprecipitation (IP) of protein extracts from agroinﬁltrated leaves using GFP-Trap conﬁrmed that GFP-tagged StLRPK1 speciﬁcally associated with StSERK3A-cMyc and not with the cMyc-StBSL1 control. The expression of constructs in the leaves is indicated by +. Protein size markers are indicated in kD, and protein loading is indicated by Ponceau stain (PS). (B) IP of protein extracts from agroinﬁltrated leaves using GFP-Trap conﬁrmed that GFP-tagged StLRPK1 associated with StSERK3A-cMyc with (+) or without (–) infiltration of *P. infestans* culture filtrate (CF). The expression of constructs in the leaves is indicated by +.

A co-immunoprecipitation assay in *N. benthamiana* was undertaken to test the potential association of StLRPK1 with the co-receptor StSERK3A/BAK1 *in planta*. StLRPK1-GFP was co-expressed with cMyc-tagged StSERK3A/BAK1 (StSERK3A/BAK1-cMyc) or with cMyc-tagged StBSL1 (a brassinosteroid phosphatase, as a non-interacting control; Saunders *et al.*, 2012) and then pulled down with GFP-Trap beads. StLRPK1-GFP, StSERK3A/BAK1-cMyc, and cMyc-StBSL1 were all stable when transiently expressed *in planta*, as indicated in the input samples. Fig. 6A shows that, although all proteins were present in the relevant input samples, StLRPK1-GFP was immunoprecipitated only with StSERK3A/BAK1-cMyc but not with the cMyc-StBSL1 control. We also confirmed that StLRPK1 interacts with NbSERK3A/BAK1 *in planta* (Supplementary Fig. 4). In summary, the co-immunoprecipitation experiments provide evidence that StLRPK1 constitutively associates with StSERK3A/BAK1 and NbSERK3A/BAK1, although further experiments are required to investigate whether this is a direct interaction leading to transphosphorylation.

It has been shown that SERKs usually dimerize with receptors upon perception of the cognate ligand (Han *et al.*, 2014; Ma *et al.*, 2016). SERK3A/BAK1 associates with receptors BRI1 and FLS2 only when they bind to their cognate ligands BR and flg22, respectively (Santiago *et al.*, 2013; Sun *et al.*, 2013). The tobacco COLD SHOCK PROTEIN (CSP) RECEPTOR (CSPR) associates with SERK3A/BAK1 upon csp22 treatment (Saur *et al.*, 2016). A similar mechanism of complex formation and activation also extends to the Pep1-induced association of PEPR1 with SERK3A/BAK1 (Tang *et al.*, 2015) and IDA-induced association of HAESA with SERK1 (Santiago *et al.*, 2016). The secreted EPIDERMAL PATTERNING FACTOR (EPF) ligands induce SERK interactions with ERECTA and the close homolog ERL1 (Meng *et al.*, 2015). However, there are examples of SERKs associating with LRR-RLPs in a ligand-independent manner. The LRR-RLP TOO MANY MOUTHS (TMM) constitutively associates with ERECTA and ERL1 to regulate stomatal patterning (Lee *et al.*, 2012). TMM also associates with SERKs, but in an EPF-independent manner (Meng *et al.*, 2015). In this study, we found that StLRPK1-GFP could immunoprecipitate StSERK3A/BAK1-cMyc in *N. benthamiana* without CF induction, indicating that the association of StLRPK1 with StSERK3A/BAK1 may occur in a ligand-independent manner (Fig. 6B). Similarly, ELR associates with potato StSERK3A/BAK1 independently of INF1 treatment (Du *et al.*, 2015). Moreover, Wang *et al.* (2018) reported that RXEG1 (*Nicotiana* LRR receptor-like protein that responds to *Phytophthora sojae* MAMP XEG1) interacts with SERK3A/BAK1 *in planta* even without XEG1 elicitation. Nevertheless, XEG1 treatment can significantly promote the RXEG1-SERK3A/BAK1 interaction. In the present study, we observed that *StLRPK1* transcripts accumulate in response to *P. infestans* CF. However, CF likely contains many MAMPs (McLellan *et al.*, 2013), and the potential specific ligands/MAMPs required for StLRPK1-mediated immunity have not yet been identified. PRRs represent a means by which broad-scale resistance can be enhanced (Tang *et al.*, 2017). However, although some PRRs have been identified that detect bacterial PAMPs, very few have yet been discovered that are responsible for detecting PAMPs from the major pests and pathogens of plants (oomycetes, fungi, nematodes, and insects). Up to now, only the RLP23, detecting NLPs (Albert *et al.*, 2015), and receptor ELR, detecting elicitons (Du *et al.*, 2015), have been shown to detect conserved molecules from oomycete pathogens.

Identification of MAMPs and the PRRs through which they activate immunity remains a major challenge (Ma *et al.*, 2016). To unravel the role of StLRPK1 as a positive regulator of immunity, future work will focus on identifying its potential ligands/MAMPs and the detailed molecular mechanisms of the StLRPK1-StSERK3A/BAK1 association.

### StLRPK1 requires NbSERK3A/BAK1 to inhibit *P. infestans* colonization

As a common co-receptor, SERK3A/BAK1 is a central regulator of innate immunity in plants via its interactions with other receptors to form the receptor complex for signaling activation. SERK3A/BAK1 is required for ligand-triggered hypersensitive response and resistance. For example, NbSERK3A/BAK1 is required for *N. benthamiana* resistance to *P. infestans* (Chaparro-Garcia *et al.*, 2011). The LRR-RLP ELR from the wild potato *Solanum microdontum* is a receptor of INF1. ELR is required for the defense responses triggered by INF1, a secreted elicitin from *P. infestans*. INF1-triggered defense responses depend on NbSERK3A/BAK1 in *N. benthamiana* (Du *et al.*, 2015).

**Fig. 7.**
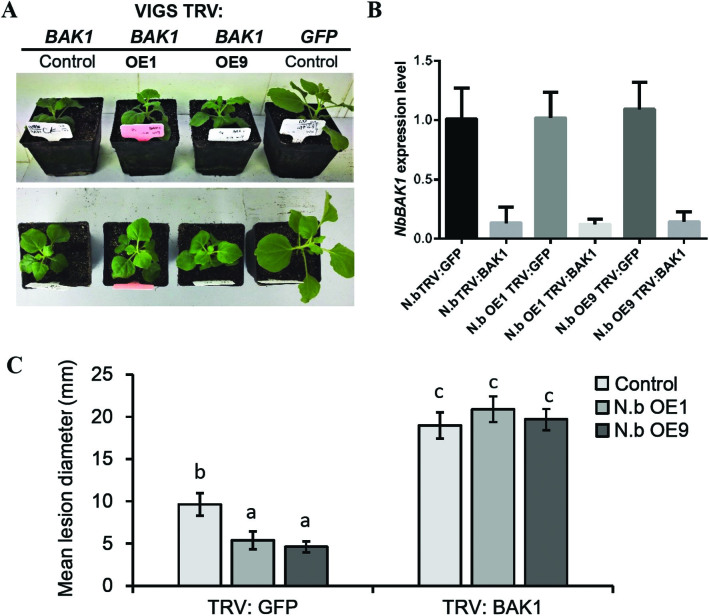
Silencing of *NbBAK1* compromises the ability of StLRPK1 to inhibit *P. infestans* colonization in *N. benthamiana*. (A) Representative image of *N. benthamiana* plants after VIGS of *NbBAK1*. OE1 and OE9 are two transgenic homozygous lines ectopically overexpressing *StLRPK1*. (B) Silencing efficiency is shown by the mean fold change measured by qRT-PCR of three biological replicates, using *N. benthamiana* plants 3 weeks post-inoculation with TRV:GFP and TRV:BAK1 vectors. (C) Mean *P. infestans* lesion diameter measured at 6 dpi in a sample of approximately 60 leaves (approximately120 lesions) for each construct in each VIGS background from four biological replicates. *P. infestans* isolate 88069 sporangia were used to inoculate leaves. Values labeled with different letters are statistically different by one-way ANOVA using pairwise multiple comparison procedures with the Holm–Sidak method (*P*<0.01). Error bars represent ±SD.

As ectopic overexpression of StLRPK1 in *N. benthamiana* significantly increased resistance to *P. infestans* (Fig. 5), and StLRPK1 interacts with the StSERK3A/BAK1 (Fig. 6), this prompted us to explore further whether StLRPK1-mediated late blight resistance is dependent on StSERK3A/BAK1. To do this, late blight resistance was measured in EV-GFP and *NbSERK3A/BAK1* VIGS plants. Typical phenotypes and qRT-PCR confirmed efficient silencing of *NbSERK3A*/*BAK1* in *N. benthamiana* plants (Fig. 7A, B). Crucially, in TRV-GFP VIGS plants, we observed that the two selected *N. benthamiana* StLRPK1 overexpression lines showed significantly smaller lesion diameters compared with those of control plants (Fig. 7C), which correlated with elevated defense levels in *N. benthamiana* plants. However, no significant differences in lesion diameters were found in the *NbSERK3A/BAK1* VIGS background plants between StLRPK1 overexpression lines and the control (Fig. 7C), indicating that StLRPK1 was no longer able to reduce *P. infestans* colonization. This confirmed that *NbSERK3A/BAK1* is essential for StLRPK1 to activate a defense response against *P. infestans*. Overall, this evidence supports a model in which StLRPK1-mediated defense depends on a protein complex containing SERK3A/BAK1.

### StLRPK1-mediated inhibition of *P. infestans* colonization relies on the MAPK pathway

Activation of PRRs usually triggers MTI via MAPK cascades, which typically contain three sequentially activated kinases: a MAPK kinase kinase (MAP3K or MEKK), a MAPK kinase (MAP2K or MKK), and a MAPK (MPK) (Pitzschke *et al.*, 2009). MAPK cascades serve as convergence points downstream of multiple cell surface-resident receptors. In Arabidopsis, the RLK FLS2 associates with BAK1 to activate the MAP3K MEKK1, which activates the MAP2Ks MKK4 and MKK5, which in turn activate the MAPKs MPK3 and MPK6 to positively activate MTI responses (Chinchilla *et al.*, 2007). In tobacco and *N. benthamiana*, the MPK6 and MPK3 orthologs salicylic acid-induced protein kinase (SIPK) (Zhang and Klessig, 1998) and wound-induced protein kinase (WIPK) are two major defense-associated MAPKs downstream of the MAP2K MKK1/MEK2 (Asai *et al.*, 2008; Seo *et al.*, 1995). VIGS of *NbSIPK* and *NbWIPK* in *N. benthamiana* abolishes antibacterial immunity, indicating that both NbWIPK and NbSIPK make important contributions to PTI in *N. benthamiana* (Segonzac *et al.*, 2011).

**Fig. 8.**
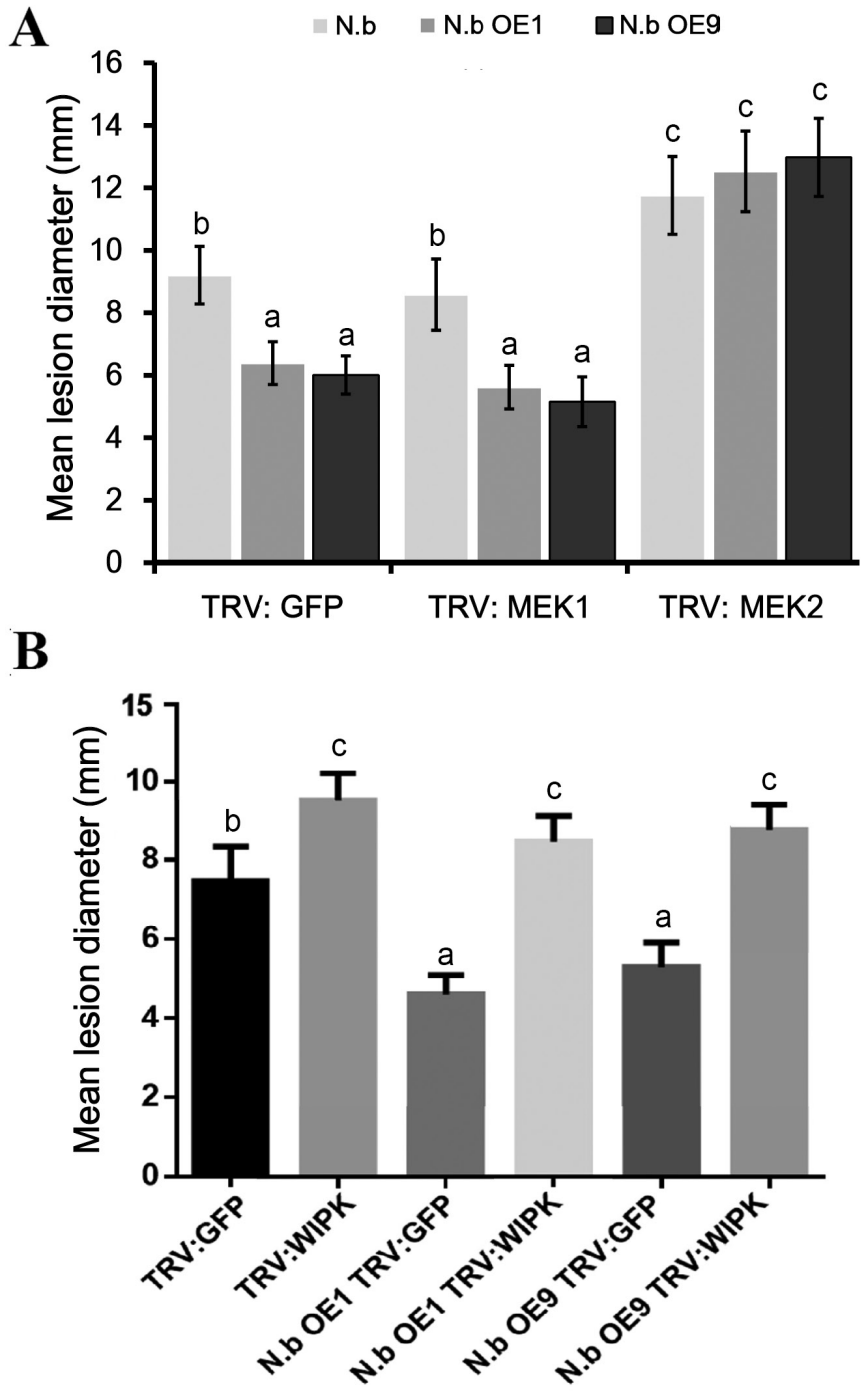
StLRPK1-mediated inhibition of *P. infestans* colonization in *N. benthamiana* depends on *NbMEK2* and *NbWIPK.* (A) Mean *P. infestans* lesion diameter measured at 6 dpi in a sample of approximately 60 leaves (approximately 120 lesions) for TRV:GFP, TRV:MEK1, and TRV:MEK2 in each VIGS background from three biological replicates. (B) Mean *P. infestans* lesion diameter measured at 6 dpi in a sample of approximately 70 leaves (approximately 140 lesions) for TRV:GFP and TRV:WIPK in each VIGS background from three or four biological replicates. *P. infestans* isolate 88069 sporangia were used to inoculate leaves. OE1 and OE9 are two transgenic homozygous lines ectopically overexpressing *StLRPK1*. Values labeled with different letters are statistically different by one-way ANOVA using pairwise multiple comparison procedures with the Holm–Sidak method (*P*<0.01). Error bars represent ±SE.

To examine whether VIGS of *MEK1*, *MEK2*, and *NbWIPK* diminishes StLRPK1-mediated resistance to *P. infestans* in *N. benthamiana*, *Agrobacterium* containing TRV:GFP and the VIGS vectors TRV:MEK1, TRV:MEK2, and TRV:WIPK was agroinfiltrated into *N. benthamiana* plants. The silencing efficiency of the targeted genes *MEK1*, *MEK2*, and *WIPK* was detected and confirmed by qRT-PCR, and the results demonstrate that VIGS of the three genes was effective (Supplementary Fig. 5). Measurements of *P. infestans* lesion size on TRV:GFP, TRV:MEK1, and TRV: MEK2-expressing *N. benthamiana* revealed that silencing of *MEK1* does not attenuate *P. infestans* resistance in *N. benthamiana* plants expressing *StLRPK1* compared with control plants. Notably, lesion sizes on the TRV:MEK2-expressing *N. benthamiana* plants were significantly larger compared with TRV: GFP VIGS plants. Moreover, unlike silencing of TRV: MEK1, the lesion sizes of two *StLRPK1* overexpression lines were the same as the control, which indicates that silencing of *MEK2* abolishes the StLRPK1-mediated resistance in *N. benthamiana* (Fig. 8A). These results suggest that StLRPK1-mediated resistance to *P. infestans* is dependent on MEK2 to activate MAPK signaling. In *NbWIPK* silencing experiments, *P. infestans* lesion diameters on TRV: NbWIPK control, StLRPK1 OE-1 and OE-9 plants, compared with the corresponding TRV: GFP plants, were all significantly larger (Fig. 8B). This result revealed that silencing of *NbWIPK* in *N. benthamiana* significantly attenuated StLRPK1-mediated *Phytophthora* resistance. These results indicate that StLRPK1-triggered *Phytophthora* resistance relies on NbWIPK for the activation of PTI.

We found that two important conserved motifs, HxD and DFG, of the catalytic core of protein kinases were replaced by HRN and DCG in two Arabidopsis SRF and StLRPK1 putative kinase domains (Fig. 1A), indicating that they are likely enzymatically inactive kinases. A number of enzymatically inactive receptor kinases are described in the literature (Kroiher *et al.*, 2001). However, signaling by enzymatically inactive kinases is poorly understood in plants (Castells and Casacuberta, 2007; Gish and Clark, 2011). They may function via regulated protein–protein interactions with downstream effectors (Kroiher *et al.*, 2001). The kinase activity is not essential for SUB function (Chevalier *et al.*, 2005; Vaddepalli *et al.*, 2011), but SUB interacts synergistically with an RLK, ERECTA, in the control of internode length (Vaddepalli *et al.*, 2011). Recently, Bai *et al.* (2013) reported that SUB interacts with ANGUSTIFOLIA directly, and ANGUSTIFOLIA is involved in SUB-dependent signaling events in Arabidopsis morphogenesis. In addition, SUB interacts in a complex with QUIRKY and PAL OF QUIRKY to regulate cell growth anisotropy during Arabidopsis gynoecium development (Trehin *et al.*, 2013). As StLRPK1 might be an enzymatically inactive receptor kinase, like SUB, there would likely be other factors acting together with or downstream of StLRPK1 to transmit signals.

In conclusion, we found that StLRPK1 localizes at the plasma membrane, where it interacts with StSERK3A/BAK1 in plant cells. We further found that StLRPK1-mediated *P. infestans* resistance in *N. benthamiana* depends on NbSERK3A/BAK1 and the MAPK signaling cascade components MEK2 and WIPK. It will be interesting to unravel this signaling pathway in detail in the future, and to identify the potential ligand(s) required for activating PTI via StLRPK1. Moreover, as RXLR effectors from *P. infestans* have been shown to block the phosphorylation and activation of WIPK following the perception of diverse pathogen elicitors (King *et al.*, 2014; Zheng *et al.*, 2014), and indeed effector PexRD2 suppresses a MEK2-dependent signal transduction pathway leading to programmed cell death (King *et al.*, 2014), it will be interesting to investigate whether specific RXLR effectors suppress the signal transduction from StLRPK1.

## Supplementary data

Supplementary data are available at *JXB* online.

Table S1. Primers and constructs used in this study.

Fig. S1. Protein alignment of the SRF3-like family proteins from Solanaceae species.

Fig. S2. The potato SRF3 isoform X1 (XP_006350044.1) does not respond to CF treatment in potato.

Fig. S3. Ectopic expression of StLRPK1 in transgenic *N. benthamiana* and homozygote screening by kanamycin resistance selection.

Fig. S4. StLRPK1 interacts with NbSERK3A *in planta*.

Fig. S5. Silencing efficiency of TRV:MEK1, TRV:MEK2, and TRV:WIPK constructs.

Supplementary Material
