# Supplementary material for: A potato STRUBBELIG-RECEPTOR FAMILY member, StLRPK1, associates with StSERK3A/BAK1 and activates immunity
Source: J Exp Bot. 2018 Aug 22;69(22):5573–86. doi: 10.1093/jxb/ery310 (PMC6255708; doi:10.1093/jxb/ery310)
Supplement: Supplementary Material [file ery310_suppl_supplementary_material.pdf]

Table S1. Primers and constructs used in this study.

Primer name	Sequencing (5'-3')		Purpose or vectors	References
StLRPK1-pBI-F	CACCCGGGATGGGTTGGAAGAGATCTG	*Sma*I	For constructing OE vector	
StLRPK1-pBI-R	TAGAGCTCTCATTCTTCATCGGATCTAC	*Sac* I	pBI121-StLRPK1	
attB1-StLRPK1-RNAi-F	AAAAGCAGGCTCCATCCAGTCTGCCAGTTACAT	attB1	For constructing RNAi vector	
attB2-StLRPK1-RNAi-R	AGAAAGCTGGGTGTGGTCAGGGAGGACAAGTT	attB2	pHellsgate8-StLRPK1	
qPCR-LRPK1-F	CTCGCAGATTTGAATGTGGA		For *StLRPK1* gene expression test	
qPCR-LRPK1-R	CGATACTACTGTTGGGTGGA			
qPCR-StSRF3-F	CTCCTTTACCTCCACCCACA		*StSRF3* Isoform X1 gene expression test	
qPCR-StSRF3-R	GGCCTGTGTTTTAGGTTTGC			
NPTⅡ-F	AGACAATCGGCTGCTCTGAT		For *NPTII* gene test	
NPTⅡ-R	TCATTTCGAACCCCAGAGTC			
StAct-F	CAGAAAGGACCTCTACGGTAACATT		Potato housekeeping gene	
StAct-R	TCTGTGGACGATGGACGGAC			
attB1-StLRPK1-F	AA AAA GCAGGCTTCATGGGTTGGAAGAGATCTGTG	attB1	C-terminal GFP fusion.<br>Fluorescence detecting. Co-IP<br>pK7FWG2.0-StLRPK1	
attB2-StLRPK1-R	AGAAAGCTGGGTCTTCTTCTTCATCGGATCTACTGG	attB2		
attB1- NbSERK3A-F	AA AAAGCAGGCTCAATGGATCAGTCGGTCTTGTTGAT		4×Myc Co-IP	
attB2- NbSERK3A-R	AGAAAGCTGGGTGTCTTGGCCCCGACAATTCATC		NbSERK3A-pGWB17	
attB1- StSERK3A-F	AA AAA GCAGGCTTCATGGATCAGTCGGTGTTGGC		10×Myc Co-IP	Nakagawa et al., 2007
attB2- StSERK3A-R	AGAAAGCTGGGTCTTCTCTTGGCCCTGACAACT		StSERK3A-pGWB20	
attB1- StBSL1-F	AA AAA GCAGGCTTCATGGGTTCAAAGCCATGG		4×Myc Co-IP	
attB2- StBSL1-R	AGAAAGCTGGGTCTTCTTAAATATAGGCAAGTGAGCT		StBSL1-pGWB18	
PiO8-3-3F	CAATTCGCCACCTTCTTCGA		*P. infestans* biomass assay	Llorente et al., 2010
PiO8-3-3R	GCCTTCCTGCCCTCAAGAAC			
StEF1a-F	ATTGGAAACGGATATGCTCCA		Potato housekeeping gene	
StEF1a-R	TCCTTACCTGAACGCCTGTCA			
TRV2-NbBAK1-F	CCGAATTCGTGAGGGTGGTGAGCGGGATAAT	*Eco*R I	VIGS construction pTRV2	Heese et al., 2007
TRV2-NbBAK1-R	CCCTCGAGGCTCATAACTGGGCAAAGGGCTT	*Xho* I		
NbBAK1-qPCR-F	TCCTGACGGACCATCTCCTCTTT		qRT-PCR for silencing efficiency test	
NbBAK1-qPCR-R	GCTCATAACTGGGCAAAGGGCTT			

NbEF1α-F	AAGGTCCAGTATGCCTGGGTGCTTGAC		*N. benthamiana* housekeeping gene	
NbEF1α-R	AAGAATTCACAGGGAC AGTTCCAATACCA			
Nb MEK1-F-*Acc*I	CCGGTCGACCTCAGAAACTAAGGAGATAGATCTT			
Nb MEK1-R-*Cla*I	CCATCGATAAAACCTGCTTGCAAACAACTG			Asai et al., 2008
Nb MEK2-F-*Cla*I	CCATCGATAGATGTGCCGTGAGATCGA		VIGS construction pTRV2	
Nb MEK2-R-*Cla*I	CCATCGATGCCTCGAGTTGATTAGTAAATTCG			
Nb WIPK-F-*Bam*HI	CGGGATCCCAGATGGTACAGGGCACCAG		VIGS construction pTRV2	
Nb WIPK-R-*Hin*dIII	CCCAAGCTTCGACAAGATCAATAGCCAATGGG			Asai et al., 2008
qRT-NbWIPK-F	GGAAGCATTAGATCATCCCTACC			
qRT-NbWIPK-R	GCTCTTCTCCTATTCCTTGTTGC			
qRT-MEK1-F	TGAGCTTCTGGAGGCTATTGTT			Melech-Bonfil and Sessa, 2010
qRT-MEK1-R	CTATCCCTTGGGTCCTTTTGTA		qRT-PCR for silencing efficiency test	
qRT-MEK2-F	GTTCAGGGACTTTATTGCTTGC			
qRT-MEK2-R	GGTAGTGGCTGGTCTATTCTGG			

StBSL1 (JQ950742); StSERK3A (KC914391); StLRPK1 (ABS89271.1)

Reference

Asai S, Ohta K, Yoshioka H. 2008. MAPK signaling regulates nitric oxide and NADPH oxidase-dependent oxidative bursts in *Nicotiana benthamiana*. The Plant Cell **20**, 1390-1406.

Heese A, Hann DR, Gimenez-Ibanez S, Jones AM, He K, Li J, Schroeder JI, Peck SC, Rathjen JP. 2007. The receptor-like kinase SERK3/BAK1 is a central regulator of innate immunity in plants. Proceedings of the National Academy of Sciences, USA **104**, 12217-12222.

Llorente B, Bravo-Almonacid F, Cvitanich C, Orlowska E, Torres HN, Flawia MM, Alonso GD. 2010. A quantitative real-time PCR method for in planta monitoring of *Phytophthora infestans* growth. Letters in Applied Microbiology **51**, 603-610.

Melech-Bonfil S, Sessa G. 2010. Tomato MAPKKKε is a positive regulator of cell-death signaling networks associated with plant immunity. The Plant Journal **64**, 379-391

Nakagawa T, Kurose T, Hino T, Tanaka K, Kawamukai M, Niwa Y, Toyooka K, Matsuoka K, Jinbo T, Kimura T. 2007. Development of series of gateway binary vectors, pGWBs, for realizing efficient construction of fusion genes for plant transformation. Journal of Bioscience and Bioengineering **104**, 34-41.

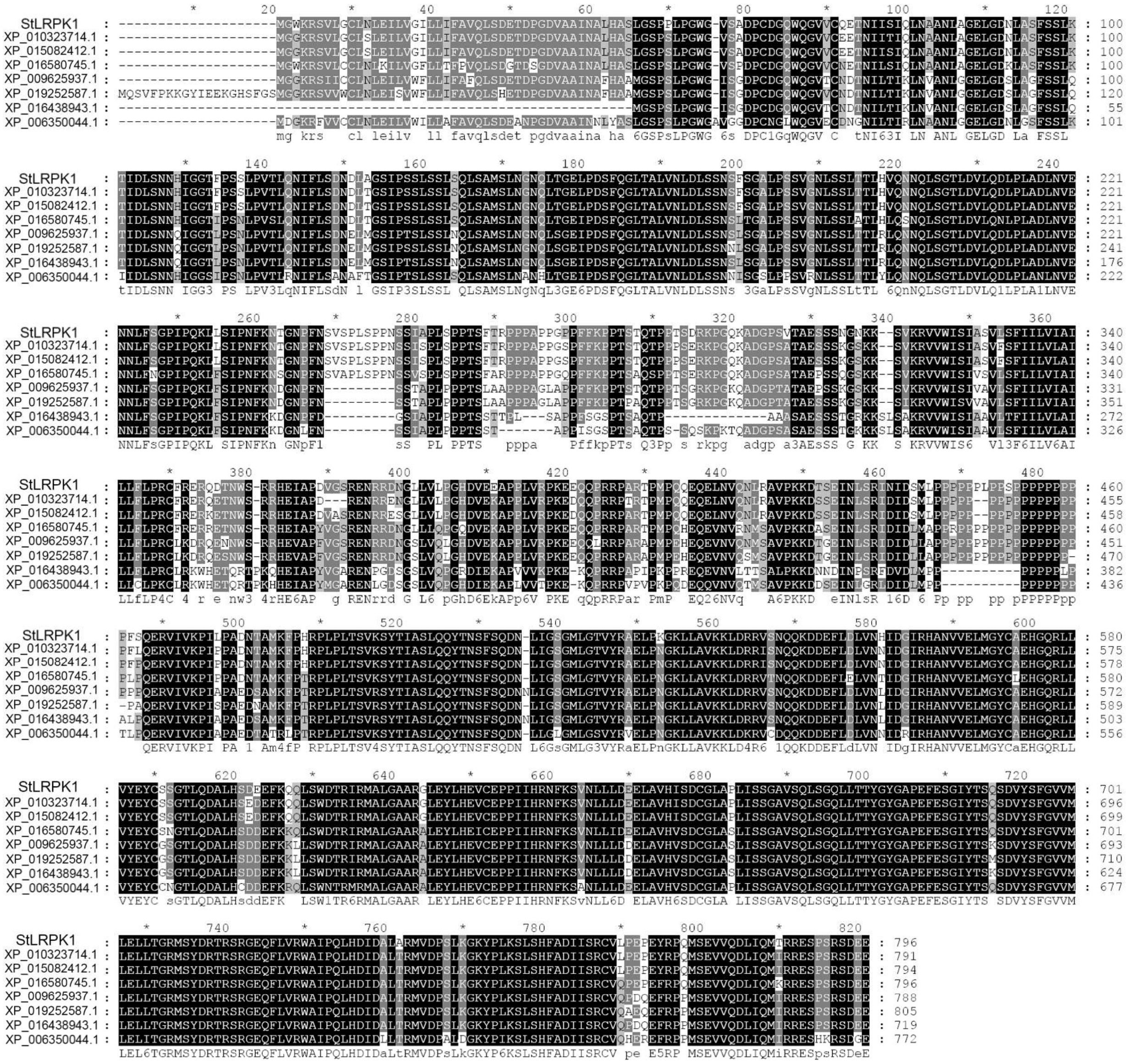

Percent Identity Matrix - created by Clustal2.1

1: XP_006350044.1	100.00							
2: XP_016580745.1	79.56	100.00						
3: StLRPK1	79.04	89.95	100.00					
4: XP_010323714.1	80.26	90.39	95.95	100.00				
5: XP_015082412.1	80.08	90.55	95.84	99.37	100.00			
6: XP_016438943.1	84.93	83.36	82.38	82.87	82.94	100.00		
7: XP_009625937.1	80.34	87.67	86.40	86.70	86.50	90.22	100.00	
8: XP_019252587.1	80.34	87.13	86.11	86.41	86.08	89.09	97.07	100.00

Fig. S1. Protein alignment of the SRF3-like family proteins from *Solanaceae* species. NP_001275442.1, StLRPK1 [*Solanum tuberosum*]; XP_006350044.1, SRF3 isoform X1 [*Solanum tuberosum*]; XP_010323714.1, SRF3-like isoform X1 [*Solanum lycopersicum*]; XP_015082412.1, SRF3-like [*Solanum pennellii*]; XP_016580745.1, SRF3 isoform X1 [*Capsicum annuum*]; XP_009625937.1, SRF3-like [*Nicotiana tomentosiformis*]; XP_016438943.1, SRF3-like [*Nicotiana tabacum*]; XP_019252587.1, SRF3-like isoform X1 [*Nicotiana attenuata*]. Protein alignment was performed by using MEGA4 software.

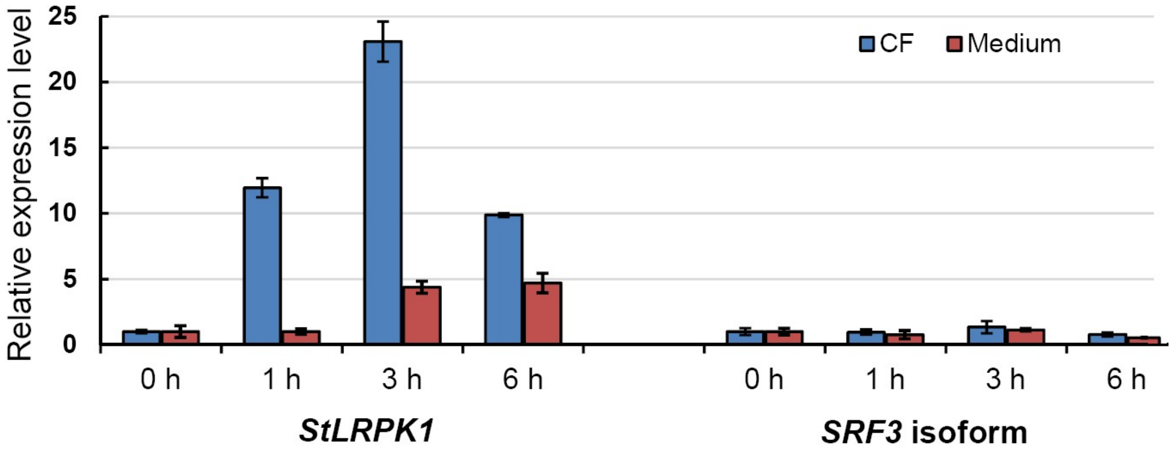

Fig. S2. The potato *SRF3* isoform X1 (XP_006350044.1) does not respond to CF treatment in potato. Potato leaves on intact plants were pressure infiltrated with CF (*P. infestans* culture filtrate) or medium control. Treated leaves were collected at 0, 1, 3 and 6 h. qRT-PCR was performed to test gene expression level. Data represents three biological replicates.

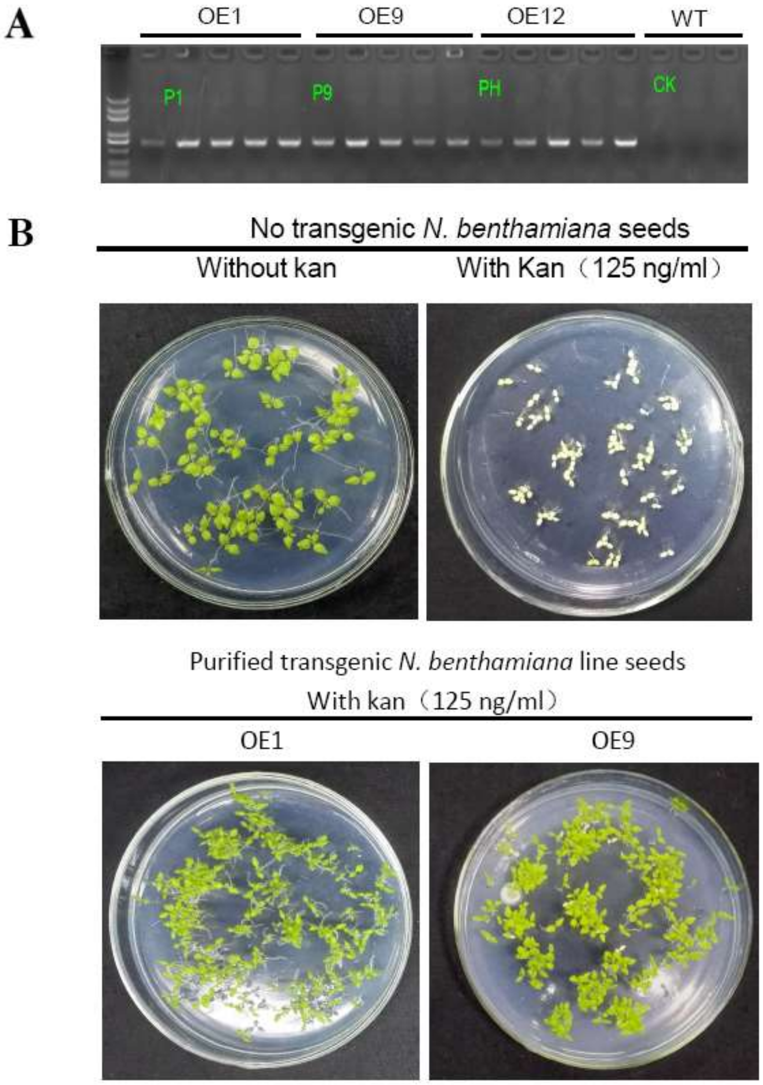

Fig. S3. Ectopic expression of *StLRPK1* in transgenic *N. benthamiana* and homozygote screening by kanamycin resistance selection. (A) Expression of *StLRPK1* in 35S: StLRK1 transgenic *N. benthamiana* lines using RT-PCR. Coding region of *StLRK1* was amplified with 32 PCR cycles. (B) Screen and identification of homozygote transgenic *N. benthamiana* lines by kanamycin (kan) resistance selection. *N. benthamiana* seeds were planted on sucrose free solid Murashige and Skoog medium containing 125 ng/mL kan.

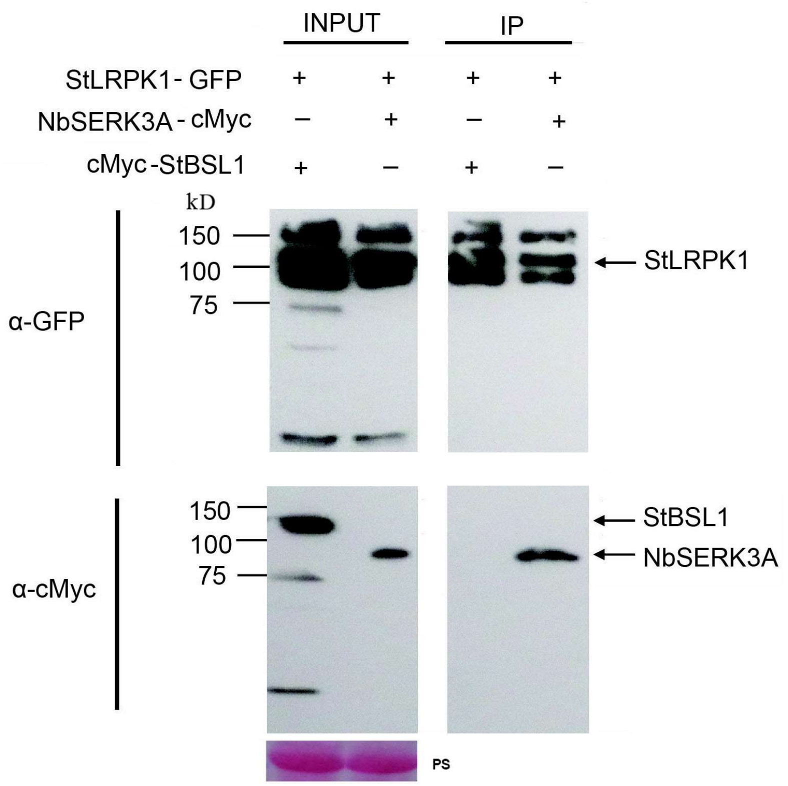

Fig. S4. StLRPK1 interacts with NbSERK3A *in planta*. Immunoprecipitation (IP) of protein extracts from agro-infiltrated leaves using GFP-Trap confirmed that GFP-tagged StLRPK1 associated with NbSERK3A-cMyc rather than cMyc-StBSL1. The expression of constructs in the leaves is indicated by +. Protein size markers are indicated in kD, and protein loading is indicated by Ponceau stain (PS).

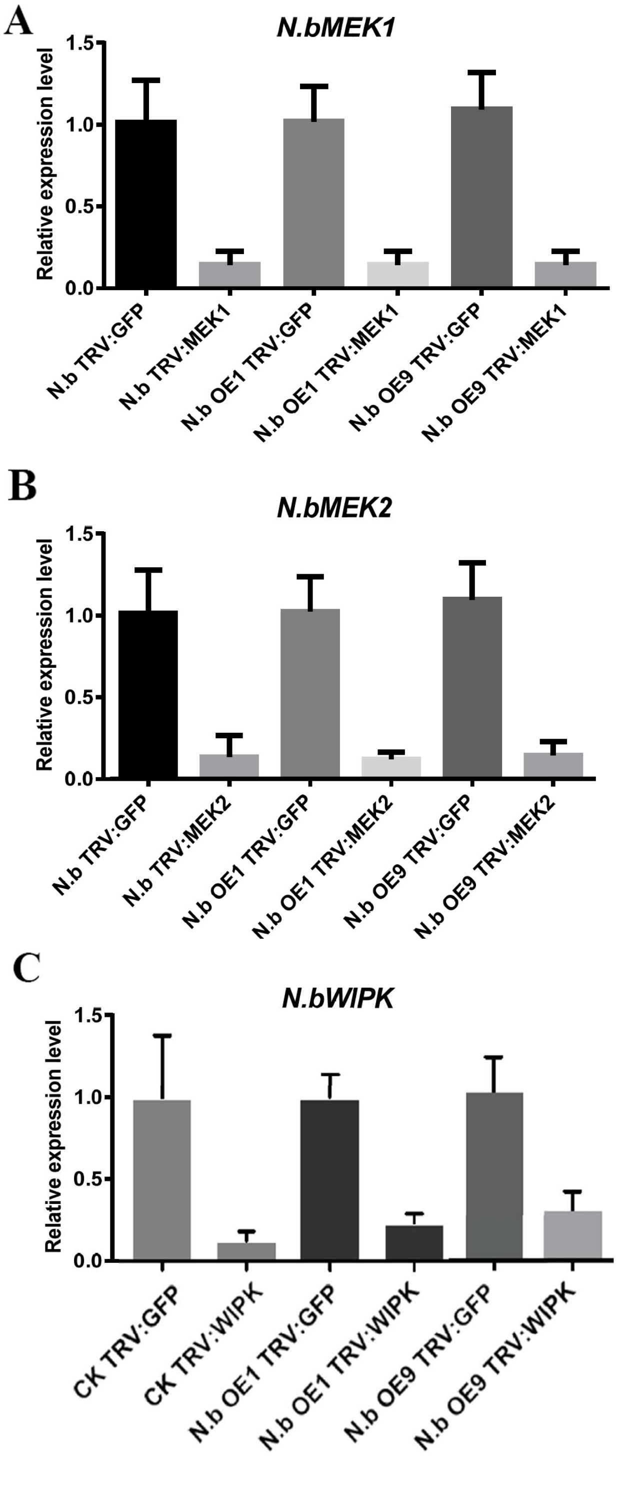

Fig. S5. Silencing efficiency of TRV: MEK1, TRV: MEK2 and TRV: WIPK constructs. Silencing efficiency shown by the mean fold change measured by qRT-PCR of 3 biological replicates using *N. benthamiana* plants 3 w post-inoculation with TRV: GFP, TRV: MEK1, TRV: MEK2 and TRV: WIPK constructs. OE1 and OE9 are two transgenic homozygote lines ectopically overexpressing *StLRPK1*. Each graph show expression of the gene indicated above it.
